# Communication at the host-pathogen interface

**DOI:** 10.1080/20002297.2017.1325269

**Published:** 2017-05-30

**Authors:** Sarah A. Kuehne

**Affiliations:** ^a^ School of Dentistry and Institute of Microbiology and Infection, College of Medical and Dental Sciences, University of Birmingham, UK

## Abstract

The oral cavity represents a finely balanced ecosystem in which a plethora of bacterial species lives in complex communities known as biofilms. To cohabitate efficiently, bacteria within these biofilms have evolved intricate systems of communication, such as quorum sensing, cooperation or competition, involving signaling, sharing of resources and/or metabolic activities. Dysbiosis can develop and lead to periodontitis, the most common human chronic inflammatory disease. Briefly, Streptococci, recognised as early colonisers, are followed by *Fusobacterium nucleatum*, known as a ‘bridging organism’. The latter interacts with multiple bacterial species, facilitating their adherence, leading to formation of a disease-associated biofilm. Specifically, bacteria of the “red complex” (*Porphyromonas gingivalis, Tannerella forsythia, Treponema denticola*) and *Aggregatibacter actinomycetemcomitans* emerge to prominence within the periodontal pocket.

While this aggregation of different species is well documented, their means of communication and in particular their mechanisms of recruitment are less well understood. The role of *F. nucleatum* is of particular interest as it interacts with multiple species in the oral biofilm and modulates progression from health to disease. Its role in controlling oral health could be pivotal.

Here we are investigating the impact of *F. nucleatum* in establishing the disease-associated biofilm by analysing bacterial cell-cell communication and bacteria-host interactions.

